# Three new species in the leafhopper tribe Drabescini (Hemiptera, Cicadellidae, Deltocephalinae) from southern China

**DOI:** 10.3897/zookeys.846.34003

**Published:** 2019-05-16

**Authors:** Zhou Yu, Mick Webb, Ren-huai Dai, Mao-fa Yang

**Affiliations:** 1 Institute of Entomology, Guizhou University; The Provincial Key Laboratory for Agricultural Pest Management Mountainous Region, Guiyang, Guizhou 550025, China Guizhou University Guiyang China; 2 Department of Entomology, The Natural History Museum, Cromwell Road, London SW7 5 BD, UK The Natural History Museum London United Kingdom

**Keywords:** China, Drabescini, morphology, taxonomy

## Abstract

Three new species of the leafhopper tribe Drabescini: *Drabescusbilaminatus***sp. n.**, *Drabescusmultipunctatus***sp. n.**, and *Paraboloponarobustipenis***sp. n.** are described and illustrated from southern China. A key and checklist to the species of *Parabolopona* are also provided.

## Introduction

In a review of the largest leafhopper subfamily, Deltocephalinae, Zahniser and Dietrich (2013) partly followed Dmitriev (2004) and included two groups previously included in Selenocephalinae, i.e., Drabescina and Paraboloponina as subtribes of Drabescini. Both groups have nymphs with long appendages on the pygofer (that do not persist into the adult stage). However, Drabescina are large, robust, the body mainly black or grey, antennal ledges are very strong, and frontoclypeus has a striate or rugose texture (that persists into the adult stage). Therefore, in the adult stage these characters are the main features to separate the two groups while the transverse striations or carinae on the fore margin of the head distinguish the two groups from most other leafhoppers. Since a revision of the two groups by Zhang and Webb (1996) several new taxa have been described, mainly from China. In the current work a further two new species of *Drabescus* Stål and one new species of *Parabolopona* Matsumura, from China, are described and illustrated.

## Materials and methods

Specimens were collected by sweep net. The external morphology was illustrated and described under a stereo microscope of Olympus SZX7. The images of adults were taken with a system of KEYENCE VHX-1000. Genitalia were drawn with Adobe Illustrator CS6 and Adobe Photoshop CS6.

Male genitalia were prepared by placing in the boiling solution of 8–10% NaOH for 1–2 min or in the cold solution for 12 hr, rinsed 1–2 times in the fresh water, then transferred into glycerine on glass slides for examination and dissection under an Olympus SZX7 stereo microscope. The structures of genitalia and abdomen were placed into fresh glycerine and stored in micro vials along with the specimens for the further examination.

The specimens studied are deposited in the Institute of Entomology, Guizhou University, Guiyang, Guizhou, China (**GUGC**) except where indicated.

## Taxonomy

### Drabescus

Taxon classificationAnimaliaHemipteraCicadellidae

Stål, 1870

#### Type species.

*Bythoscopusremotus* Walker, 1851

#### Diagnosis.

Overall coloration brown to black often with contrasting yellow marking on head and thorax. Body more or less robust, wedge-shaped. Crown short and broad, with transverse ridge on front, the latter slightly arched forward. Ocelli marginal, distant from eye. Face with antenna situated above midline of eye, moderately long (very long in immature); antennal ledge strong; anteclypeus nearly triangular, broad at base; laterofrontal sutures extended to corresponding ocellus. Hind femur with apical setae 2+1, 2+1+1, or 2+2+1. Male pygofer side with or without macrosetae and with or without a posterior process or marginal serrations. Connective usually Y-shaped. Subgenital plate triangular or elongate with digitate apex, usually with short fine setae marginally on ventral surface. Aedeagus with or without basal processes; gonopore apical on ventral surface.

#### Remarks.

*Drabescus* is the largest genus in the subtribe Drabescina containing 60 species in the Old World tropics of which 34 species are from China (mainly southern China).

### Drabescus bilaminatus sp. n.

Taxon classificationAnimaliaHemipteraCicadellidae

http://zoobank.org/660BE687-C6E3-453A-9647-A4ECBC39A452

Figs 1–9

#### Diagnosis.

Overall colour yellowish brown with numerous dark spots on the forewings. Subgenital plate wrinkled at apex. Aedeagal shaft with large flange on each side of ventral surface extending sub-basally to near apex.

#### Description.

Vertex approximately 1.3x as long medially than next to eyes. Ocelli separated by ca. 4 x own diameter from adjacent eye. Hind femur with apical setae 2+2+1.

Male genitalia. Pygofer side nearly quadrilateral with long stout serrated ventral process directed dorsally; without macrosetae (Fig. 4). Valve triangular, nearly 2 x as wide as medial length. Subgenital plate elongate triangular with very short, wrinkled at apex; with short fine setae marginally on ventral surface (Fig. 5). Connective with stem short, 1/2 long as arms (Fig. 9). Style slender throughout length, without distinct lateral lobe (Fig. 6). Aedeagal shaft elongate, cylindrical, evenly curved dorsally, with large flange on each side of ventral surface extending sub-basally to near apex; dorsal surface with few fine teeth (Figs 7, 8).

**Figures 1–9.**
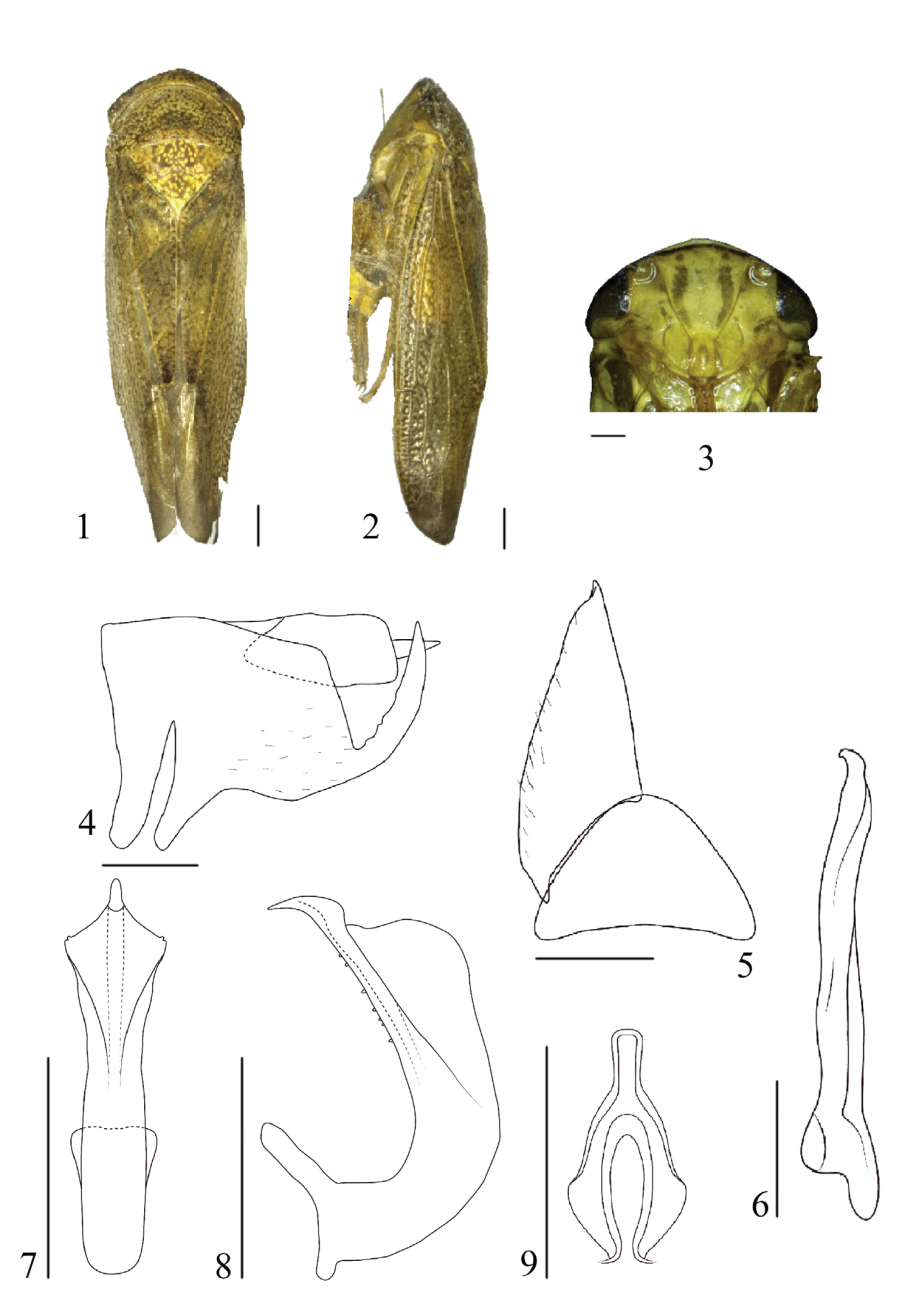
*Drabescusbilaminatus* sp. n. holotype **1** dorsal view **2** lateral view **3** face **4** pygofer, lateral view **5** valve and subgenital plate, ventral view **6** style, ventral view **7** aedeagus, ventral view **8** aedeagus, lateral view **9** connective, ventral view. Scale bars: 1.0 mm (**1, 2**); 0.5 mm (**3–9**).

**Length (including tegmen).** ♂, 11.6 mm.

#### Material examined.

Holotype: ♂, CHINA: Guangxi province, Huaping National Nature Reserve, 18.V.2012, Zhi-hua Fan leg. Paratype: 1 ♂, data same as holotype.

#### Remarks.

This new species is similar to *D.ineffectus* (Walker) but can be distinguished by its larger lateral flanges of the aedeagus, narrower style and shorter stem of the connective.

#### Etymology.

The new species name is an adjective derived from a combination of the Latin words *bi* and *lamina*, referring to the laminate (thin) flanges on the aedeagus.

#### Distribution.

China (Guangxi Province).

### Drabescus multipunctatus sp. n.

Taxon classificationAnimaliaHemipteraCicadellidae

http://zoobank.org/662F3FA6-93BC-4F7F-8EF5-B2AC246BF1E7

Figs 10–18

#### Diagnosis.

**Figures 10–18.**
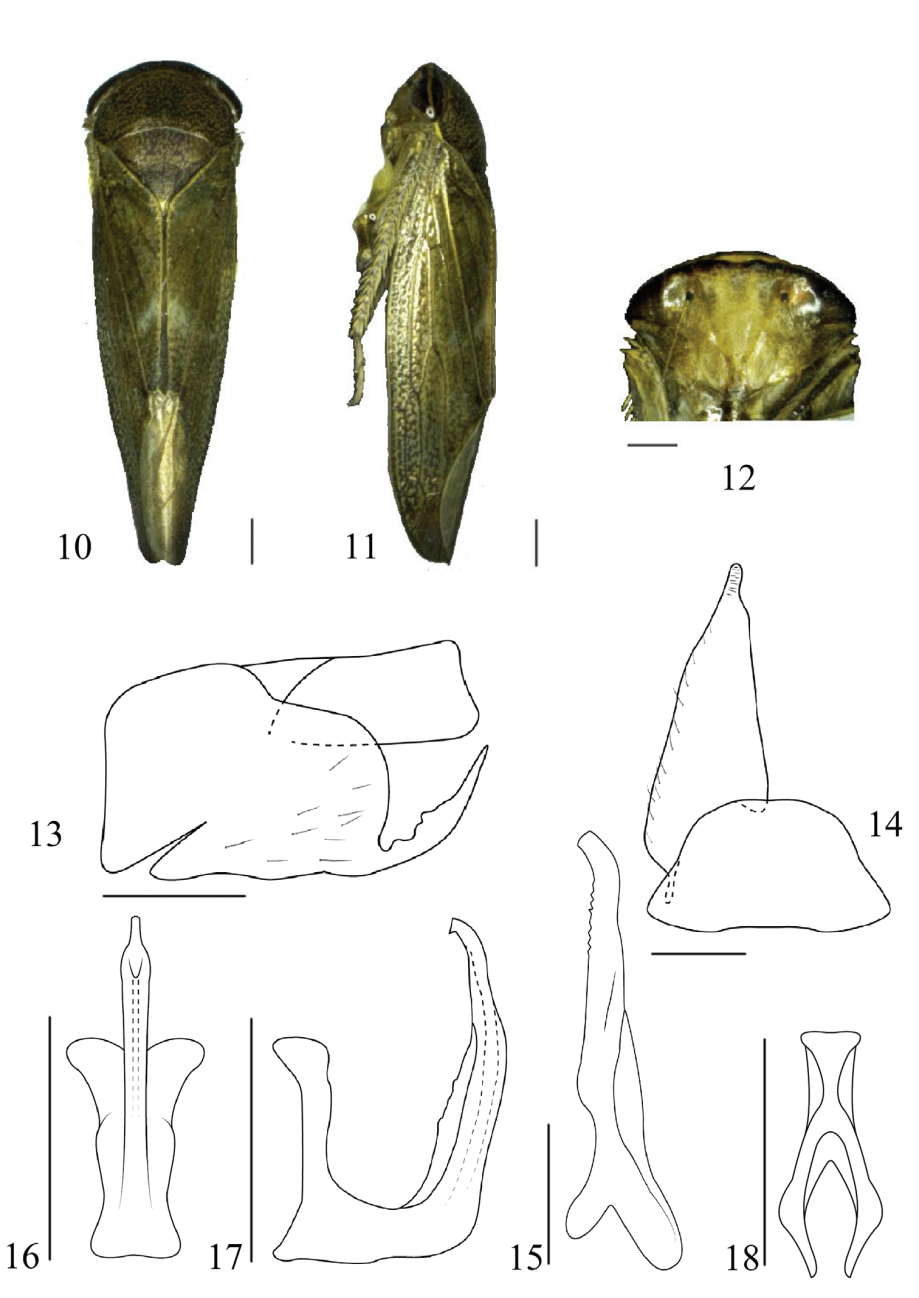
*Drabescusmultipunctatus* sp. n. holotype **10** dorsal view **11** lateral view **12** face **13** pygofer, lateral view **14** valve and subgenital plate, ventral view **15** style, ventral view **16** aedeagus, ventral view **17** aedeagus, lateral view **18** connective, ventral view. Scale bars: 1.0 mm (**10, 11**); 0.5 mm (**12–18**).

Overall colour yellowish brown with numerous fine dark spots; costal area of forewing yellow (Fig. 10). Pygofer side with long stout serrated ventral process directed dorsally. Aedeagal shaft with a long single crest-like dorsomedial flange on the dorsal surface (Figs 16, 17).

#### Description.

Vertex approximately as long as next to eyes. Ocelli marginal, situated between two transverse ridges, separated by ca. 3 x own diameter from adjacent eye (Fig. 10). Hind femur with apical setae 2+2+1.

Male genitalia. Pygofer side slightly longer than wide; apically evenly rounded except for long stout serrated ventral process directed dorsally; without macrosetae (Fig. 13). Valve semicircular, approximately 2 x as wide as medial length. Subgenital plate elongate, triangular with short digitate apex, with short fine setae marginally on ventral surface (Fig. 14). Connective with stem as long as arms (Fig. 18). Style relatively slender throughout length, lateral lobe absent, apex curved inward with few serrations subapically on inner surface (Fig. 15). Aedeagal shaft elongate, cylindrical, sharply turned dorsally sub-basally, dorsal surface with a long single crest-like dorsomedial flange (Figs 16, 17).

**Body length (including tegmina).** ♂, 10.7mm.

#### Material examined.

Holotype: ♂, CHINA: Hainan Province, Jianfeng ridge, Nanwang forest, 22.IV.2014, Wei-cheng Yang leg.

#### Remarks.

The new species can be distinguished by the shape of the aedeagus with abruptly angled shaft sub-basally and with a long single crest-like dorsomedial flange.

#### Etymology.

The new species name is an adjective derived from the Latin words *multi* and *punctatum* referring to the many fine dark spots on the body.

#### Distribution.

China (Hainan Province).

### Parabolopona

Taxon classificationAnimaliaHemipteraCicadellidae

Matsumura, 1912

Parabolopona Matsumura, 1912: 288; Webb, 1981: 41; Zhang and Webb 1996: 19; Cai and Shen 1999: 28; Shang and Zhang 2007: 430; Dai et al. 2016: 394.

#### Type species.

*Paraboloponaguttata* Uhler, 1896

#### Diagnosis.

Body yellow to yellowish green, with or without pair of orange bands on vertex and pronotum; forewings with few small brown spots. Head with anterior margin rim-like; vertex approximately twice as long medially than next to eyes with fore margin obliquely rounded, shagreen. Face with antenna situated near upper corner of eye; antennal ledge strong, antennal pits encroaching onto postclypeus; latero-frontal sutures extended to corresponding ocellus; anteclypeus rectangular. Pronotum as wide as crown with many fine transverse striations. Hind femur with apical setae 2+2+1. Male pygofer without processes. Valve nearly triangular. Subgenital plate triangular or semicircular with fine ventral setae. Connective Y-shaped with strongly produced stem apex; separated from aedeagus by membrane. Aedeagus with or without basal apodeme; shaft relatively short with or without processes, gonopore apical on ventral surface. Second valvulae with very fine dorsal teeth.

#### Remarks.

*Parabolopona* is one of several genera in the subtribe Paraboloponina. The genus contains eleven species, of which ten have been recorded from China (see checklist below).

### Checklist of genus *Parabolopona*

**Figures 19–38.**
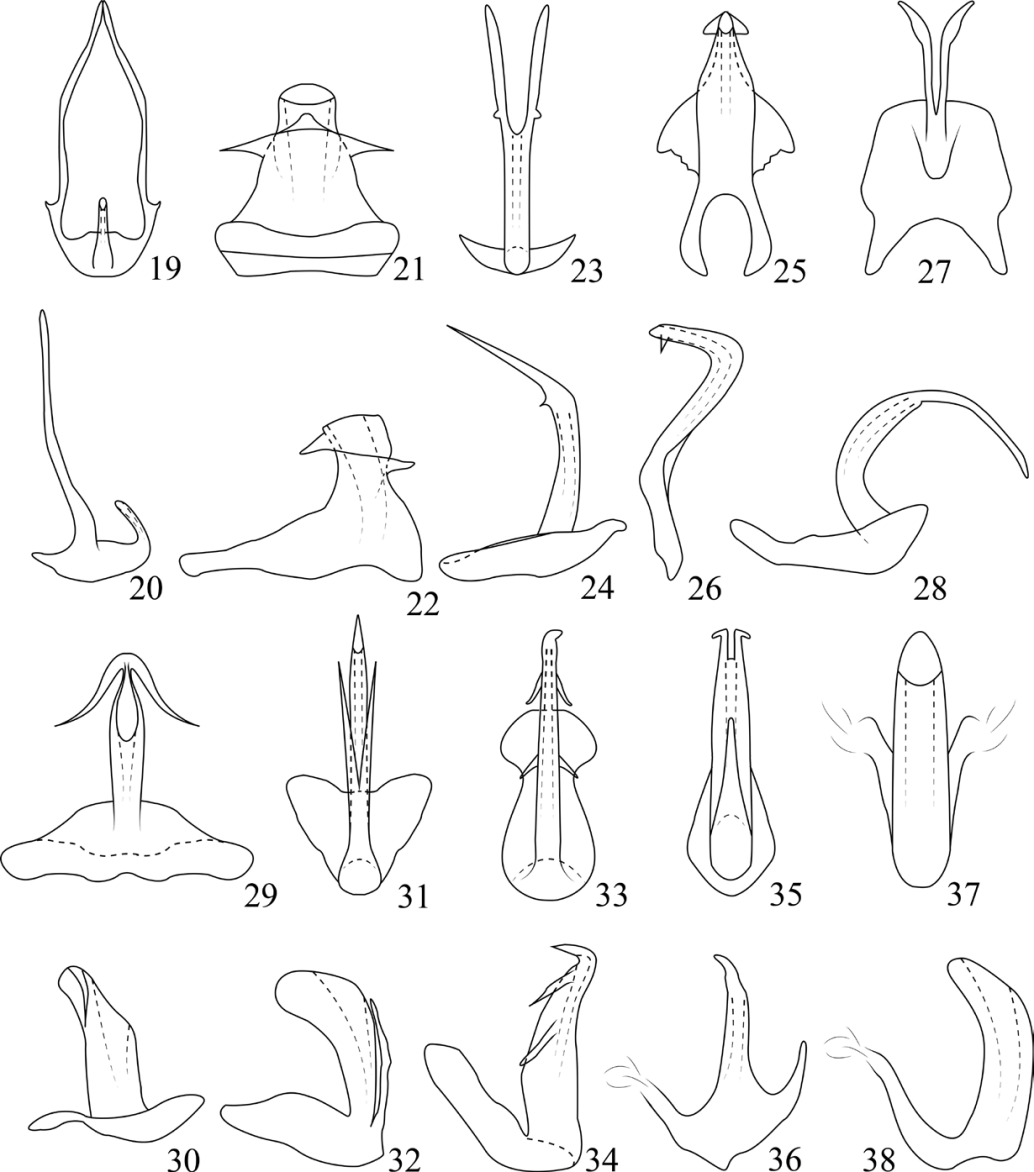
*Parabolopona* species **19, 20***P.basispina*, aedeagus, ventral and lateral view **21, 22***P.robustipenis* sp. nov., aedeagus, ventral and lateral view **23, 24***P.chinensis*, aedeagus, ventral and lateral view **25, 26***P.cygnea*, aedeagus, ventral and lateral view **27, 28***P.guttata*, aedeagus, ventral and lateral view **29, 30***P.ishihari*, aedeagus, ventral and lateral view **31, 32***P.luzonensis*, aedeagus, ventral and lateral view **33, 34***P.quadrispinosa*, aedeagus, ventral and lateral view **35, 36***P.webbi*, aedeagus, ventral and lateral view **37, 38***P.yangi*, aedeagus, ventral and lateral view.

*P.basispina* Dai, Qu & Yang, 2016: 394. Figs 19, 20. China (Hainan).

*P.robustipenis* sp. n. Figs 21, 22. China (Hainan).

*P.chinensis* Webb, 1981: 45. Figs 23, 24. China (Hubei, Sichuan, Shanxi).

*P.cygnea* Cai & Shen, 1999: 28. Figs 25, 26. China (Henan, Guizhou, Shanxi).

*P.guttata* (Uhler, 1896: 291). Figs 27, 28. Japan, Philippines, China (Taiwan).

*P.ishihari* Webb, 1981: 45. Figs 29, 30. Japan, China (Shanxi, Yunnan, Hubei).

*P.luzonensis* Webb, 1981: 46. Figs 31, 32. Philippines, China (Zhejiang, Guizhou).

*P.mutabilis* Ohara & Kogure, 2012: 205. Figs 10, 14. Japan.

*P.quadrispinosa* Shang & Zhang, 2006: 33. Figs 33, 34. China (Yunnan, Guangxi).

*P.webbi* Zahniser & Dietrich, 2013: 181. Figs 35, 36. China (Taiwan).

*P.yangi* Zhang, Chen & Shen, 1995: 11. Figs 37, 38. China (Guangdong).

*P.zhangi* Meshram, Shashank & Srinivasa, 2016: 184, 185. Figs 2–22. India.

### Key to species of *Parabolopona* (males)

**Table:** 

1	Aedeagus without processes (Figs 37, 38)	*** P. yangi ***
–	Aedeagus with processes	**2**
2	Aedeagal shaft very short and robust in both lateral and ventral view (Figs 21, 22)	***P.robustipenis* sp. n.**
–	Aedeagal shaft not very short and robust in both lateral and ventral view	**3**
3	Aedeagal shaft with two pairs of processes (Figs 33, 34)	*** P. quadrispinosa ***
–	Aedeagal shaft with one pair of processes	**4**
4	Aedaegal processes very long, arising from base of shaft	**5**
–	Aedaegal processes short to moderately long, arising ventrally from base of shaft or midlength	**6**
5	Aedeagal shaft broad distally in lateral view (Figs 19, 20)	*** P. basispina ***
–	Aedeagal shaft narrow distally in lateral view	*** P. zhangi ***
6	Aedeagal with processes arising near midlength, closely attached to each other	*** P. mutabilis ***
–	Aedeagal processes divergent, arising from base of shaft	**7**
7	Aedaegal shaft broadened apically in lateral view (Figs 31, 32)	*** P. luzonensis ***
–	Aedaegal shaft not broadened apically in lateral view	**8**
8	Apex of aedaegal shaft branched	**9**
–	Apex of aedaegal shaft unbranched	**11**
9	Aedeagal shaft straight in lateral view (Figs 35, 36)	*** P. webbi ***
–	Aedeagal shaft evenly curved in lateral	**10**
10	Connective straight and narrow apically (Figs 23, 24)	*** P. chinensis ***
–	Connective expanded apically (Figs 27, 28)	*** P. guttata ***
11	Aedeagal shaft with a pair of lateral triangular flanges in ventral view (Figs 25, 26)	*** P. cygnea ***
–	Aedeagal shaft without flanges (Figs 29, 30)	*** P. ishihari ***

### Parabolopona robustipenis sp. n.

Taxon classificationAnimaliaHemipteraCicadellidae

http://zoobank.org/80A10A6D-B4FB-491F-91C7-FA7E1B97EE0E

Figs 39–48

#### Diagnosis.

Body yellowish green. Vertex and pronotum with a pair of orange longitudinal bands. Forewing with few small dark brown spots. Connective with stem about four times longer than arms, with strong dorsal keel, apical extension long. Aedeagus with shaft very short and robust (Fig. 45).

#### Description.

**Figures 39–48.**
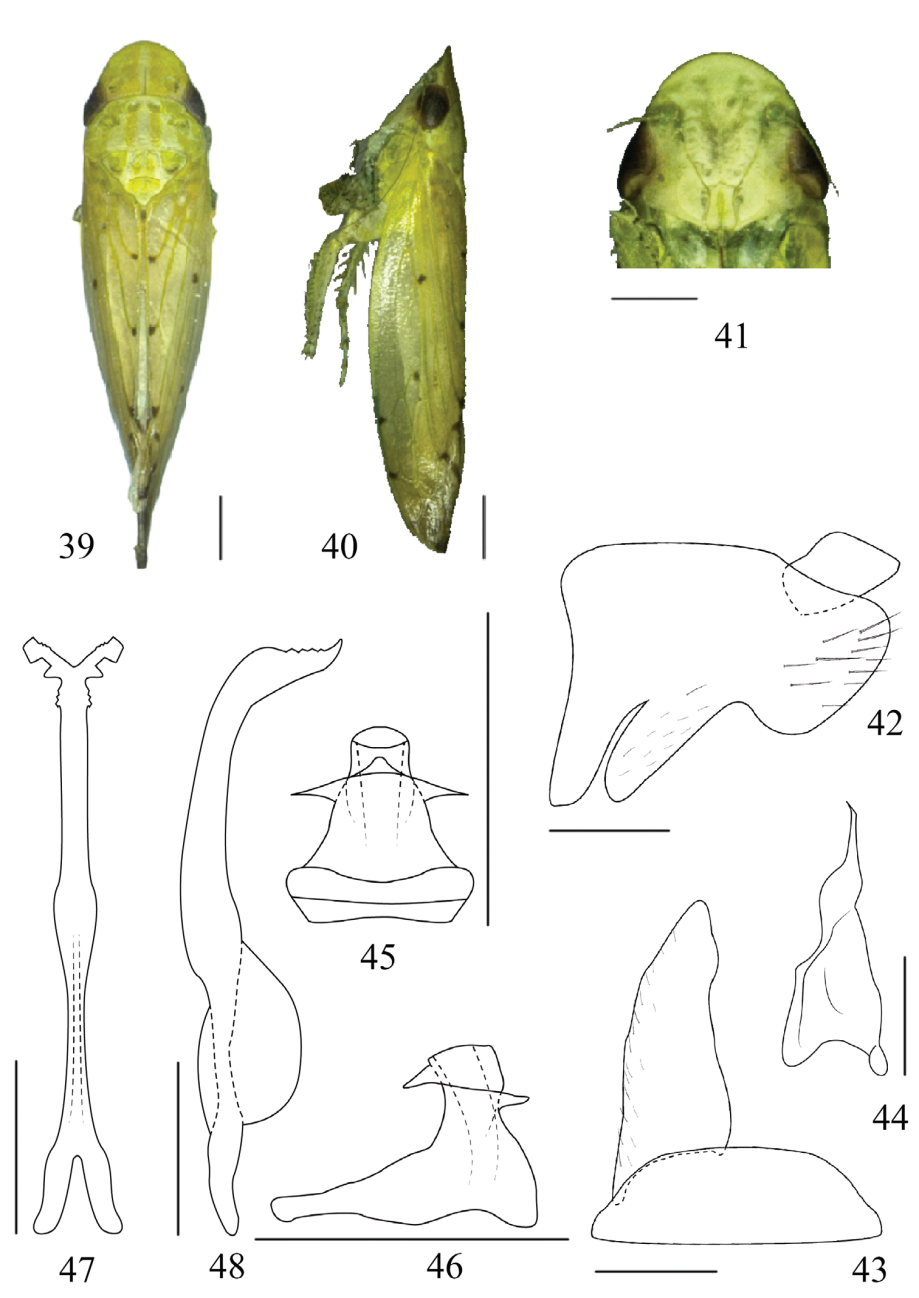
*Paraboloponarobustipenis* sp. n. holotype **39** male dorsal view **40** male lateral view **41** male face **42–48** male genitalia **42** pygofer, lateral view **43** valve and subgenital plate, ventral view **44** style, ventral view **45** aedeagus, ventral view **46** aedeagus, ventral view **47** connective, ventral view **48** connective, lateral view. Scale bars: 1.0 mm (**39, 40**); 0.5 mm (**41–48**).

Vertex approximately 2 x as long medially than next to eyes (Fig. 39). Ocelli marginal, separated by ca. 2 x own diameter from adjacent eye. Hind femur with apical setae 2+2+1.

#### Male genitalia.

Pygofer side with ventral margin strongly indented; with several fine setae distally (Fig. 42). Valve semicircular, approximately 3 x as wide as medial length. Subgenital plate elongate triangular with inner margin sinuate (Fig. 43). Connective with stem about four times longer than arms, with strong dorsal keel, apical extension long, bifurcate apically with upturned serrated branches (Figs 47, 48). Style apical process very slender, tapered to acute apex; lateral lobe prominent (Fig. 44). Aedeagus with shaft very short and robust (Fig. 45), with a short triangular shaped process subapically on dorsal surface and a lamellate processes on each side subapically, gonopore apical (Fig. 46).

#### Length

**(including tegmen).** ♂, 8.4 mm; ♀, 8.2–8.6 mm.

#### Material examined.

Holotype: ♂, CHINA: Hainan Province, Donger station, Bawang ridge, 15.IV.2014, collected by Jian-kun Long and Hai-yan Sun. Paratypes: 3 ♂♂, data same as holotype; 16 ♂♂ 2♀♀, Donger station, Bawang ridge, Hainan province, 22.IV.2014, Wei-cheng Yang, Hai-yan Sun leg. (GUGC and The Natural History Museum, London).

#### Remarks.

The new species differs from other species of the genus in the shape of the male genitalia, particularly the very short and broad aedeagal shaft with a short triangular shaped process subapically on dorsal surface and a lamellate processes on each side subapically.

#### Etymology.

The new species name is a noun derived from the Latin words *robustus* and *penis* referring to the robust aedeagus in this species.

#### Distribution.

China (Hainan province).

## Supplementary Material

XML Treatment for Drabescus

XML Treatment for Drabescus bilaminatus

XML Treatment for Drabescus multipunctatus

XML Treatment for Parabolopona

XML Treatment for Parabolopona robustipenis
